# Pentaborane: MAK Value Documentation, addendum – Translation of the German version from 2024

**DOI:** 10.34865/mb1962422e11_2ad

**Published:** 2026-06-30

**Authors:** Andrea Hartwig, MAK Commission

**Affiliations:** 1 Institute of Applied Biosciences, Department of Food Chemistry and Toxicology, Karlsruhe Institute of Technology (KIT) Adenauerring 20a, Building 50.41 76131 Karlsruhe Germany; 2 Permanent Senate Commission for the Investigation of Health Hazards of Chemical Compounds in the Work Area, Deutsche Forschungsgemeinschaft, Kennedyallee 40, 53175 Bonn, Germany. Further information: Permanent Senate Commission for the Investigation of Health Hazards of Chemical Compounds in the Work Area | DFG

**Keywords:** pentaborane, neurotoxicity, central nervous system, CNS

## Abstract

The German Senate Commission for the Investigation of Health Hazards of Chemical Compounds in the Work Area (MAK Commission) re-evaluated the occupational exposure limit value (maximum concentration at the workplace, MAK value) for pentaborane [19624-22-7] considering all toxicological end points. Relevant studies were identified from a literature search. The critical effect of pentaborane is its effect on the central nervous system (CNS). Inadequately documented studies carried out before 1960 reported findings of CNS effects in humans at concentrations below 1 ml/m^3^ and in various animal species at the lowest concentration tested of 0.2 ml/m^3^ and above (exposure period: 6 months). In rats, guinea pigs, rabbits, dogs and monkeys, mortality was also observed at this concentration. As a result, no MAK value can be derived. Pentaborane, which is a liquid at room temperature, does not penetrate the skin in toxicologically relevant amounts. There are no studies investigating developmental toxicity, genotoxicity, carcinogenicity or the sensitizing potential of pentaborane.

**Table:** 

**MAK value**	**not established, see Section II b of the List of MAK and BAT Values**
**Peak limitation**	–
	
**Absorption through the skin**	–
**Sensitization**	–
**Carcinogenicity**	–
**Prenatal toxicity**	–
**Germ cell mutagenicity**	–
	
**BAT value**	–
	
	
Synonyms	boron hydride pentaboron nonahydride
Chemical name (IUPAC)	pentaborane(9)
CAS number	19624-22-7
Molecular formula	B_5_H_9_
Molar mass	63.12 g/mol
Melting point	–46.7 °C (IFA 2002)
Boiling point	58.4 °C (IFA 2002)
Density at 0 °C	0.61 g/cm^3^ (IFA 2002)
Vapour pressure at 20 °C	213.3 hPa (Henschler 1974, available in German only) 220 hPa (IFA 2002)
log K_OW_	no data
Solubility	decomposes on contact with water (IFA 2002)
**1 ml/m^3^ (ppm) **≙** 2.619 mg/m^3^**	**1 mg/m^3^ **≙** 0.382 ml/m^3^ (ppm)**
	
Hydrolytic stability	decomposes on contact with water (IFA 2002)
Uses	catalyst, synthesis of carborane polymers, for semiconductor doping, as a fuel or fuel additive (Hart et al. 1984) corrosion inhibitor, fluxing agent, oxygen scavenger (NCBI 2005)

A MAK value of 0.005 ml/m^3^ and a peak limitation category were derived for pentaborane based on documentation published in 1974 (Henschler 1974, available in German only). No other designations or classifications were assigned.

Pentaborane is a very strong reducing agent and may ignite explosively on contact with air and other materials. Its use as a rocket propellant was therefore tested from 1950 to 1960, but found to be unworkable. However, large stocks of pentaborane from this period still existed in the 1980s. Improper storage or disposal of these stockpiles led or could potentially have led to accidents (Hart et al. 1984). A few toxicological studies were therefore carried out with the substance. Pentaborane is no longer used today.

Pentaborane undergoes an extremely exothermic reaction on contact with air, forming the more stable boron trioxide (B_2_O_3_) and water (Yarbrough et al. 1985).

## 1 Toxic Effects and Mode of Action

Pentaborane has an unpleasant odour similar to that of sour milk; however, habituation to the odour occurs. An odour threshold of 0.8 ml/m^3^ was obtained in earlier studies.

Pentaborane is corrosive to the skin and eyes. Prolonged inhalation exposure leads to irritant effects on the mucous membranes of the eyes and respiratory tract. Pentaborane has effects on the central nervous system (CNS) in humans at concentrations below 1 ml/m^3^ and in animal studies at concentrations of 0.2 ml/m^3^ and above. The severest effects with tremor, impaired mobility, incoordination and anaesthesia were observed in monkeys and dogs; mortality occurred in these species and in rabbits, golden hamsters and rats. In vitro, boron hydrides may disturb ATP synthesis and oxidative phosphorylation.

There are no data available for sensitizing effects, reproductive toxicity or genotoxic and carcinogenic effects.

## 2 Mechanism of Action

The mechanism underlying the local and central nervous effects of pentaborane is not known. Some of the local effects were probably caused by the disintegration of pentaborane on contact with an aqueous milieu to form boric acid and hydrogen (Henschler 1974) and its strong reducing potential (Hart et al. 1984).

Changes were observed in the electroencephalogram (EEG) after acute intoxication (Mindrum 1964). The effects on the CNS may be due to the inhibition of glycolysis (no other details; NCBI 2005). Boranes also lead to changes in neurotransmitter concentrations. Noradrenaline is reduced by the inhibition of synthesis; the synthesis of dopamine may be reduced as well. Serotonin levels in the brains of rats were increased (Hart et al. 1984).

Boranes reduce the absorption of oxygen, the formation of ammonia and the synthesis of glutamine in the brain tissue. They inhibit the glucose, protein and lipid metabolism in the liver tissue. Boron hydrides may disturb ATP synthesis and oxidative phosphorylation. Enzymes such as xanthine oxidase, choline oxidase, cytochrome c reductase, lactate dehydrogenase and malate dehydrogenase may be inactivated. After mild intoxication, the effects on the enzymes are reversible; however, irreversible and fatal effects may be induced at higher levels of exposure (Mindrum 1964).

## 3 Toxicokinetics and Metabolism

There are no studies available that specifically investigated this substance. Severe systemic effects were observed after accidental inhalation exposure to pentaborane (Henschler 1974; Mindrum 1964). The amount of boric acid excreted with the urine increased in dogs after inhalation exposure (Weir et al. 1964). On the basis of these findings, pentaborane is assumed to be absorbed by inhalation.

Pentaborane has a high vapour pressure (213.3 hPa) and has corrosive effects on the skin; therefore, dermal exposure to the liquid substance is unlikely. It is not possible to determine the solubility of pentaborane because it decomposes in water. As a result, models cannot be used to calculate the fraction of pentaborane in solution that is absorbed through the skin after application of non-irritant concentrations. The absorption of gaseous pentaborane was investigated in the study below.

The exposure of dogs to pentaborane concentrations of 550, 580 and 710 ml/m^3^ exclusively via the shaved skin for periods of 2, 4 and 6 hours led to delayed response times in learning tests and increased the amount of boric acid excreted with the urine by a factor of 5 (see Section 5.1.3). However, the effects caused by absorption through the skin are minor in comparison with those resulting from absorption by inhalation. Inhalation exposure to a concentration of 3.7 ml/m^3^ for only 1 hour caused convulsions, tremor and mortality. The authors of the study therefore assessed the toxicity of dermal exposure being not remarkable (Weir et al. 1964). The concentrations used were lethal for dogs after inhalation exposure (see Section 5.1.1), and the study found that the amount absorbed through the skin is lower than one percent of the amount absorbed by inhalation.

Pentaborane forms non-volatile hydrolysis intermediates (no other details) in the blood that are slowly degraded. In rats given tritium-labelled pentaborane by intraperitoneal injection, 36% molecular hydrogen formed within 3 hours after administration (NCBI 2005).

## 4 Effects in Humans

An earlier study reported an odour threshold for pentaborane of 2.5 mg/m^3^ (0.8 ml/m^3^; no other details; IFA 2002; Krackow 1953).

### 4.1 Single exposures

Acute exposure to pentaborane led to neurological and psychiatric symptoms, above all confusion, concentration disorders and short-term memory impairment (Hart et al. 1984).

A total of 154 engineers, technicians and laboratory workers were monitored by occupational physicians during a 3-year period in which they handled pentaborane. While working with pentaborane, the workers took protective measures including the use of face masks and protective clothing. Contaminated protective clothing was disposed of immediately. Nevertheless, although (emergency) medical treatment was initiated immediately, accidental exposure led to 21 severe and 46 moderate to mild cases of intoxication. In most of these accidents, a detector with a limit of detection of 1 ml/m^3^ had failed to detect pentaborane even though the odour of the substance had already been perceived. No effects were observed in the case of a “slight smell”. The persons who perceived a “strong smell” reported a penetrating sensation in the nose in addition to headaches, lethargy, confusion, difficulties concentrating, a tired and impaired appearance, changes in personality and unreasonable behaviour. Vomiting, muscle weakness, impaired speech, an increased need for sleep, restlessness, euphoria, weakness and other symptoms developed after exposure to higher concentrations. Substantial changes in the EEG confirmed these findings. After exposure to high pentaborane concentrations (no data), spasms in muscle groups, convulsions without biting of the tongue, hypoaesthesia and other symptoms developed. Some of the intoxicated persons had not perceived pentaborane. This was explained by interference through smells originating from other substances or the known effect of olfactory fatigue caused by pentaborane (Henschler 1974; Mindrum 1964).

Three persons were probably exposed to high concentrations of pentaborane in an accident that occurred during the disposal of old drums containing liquid pentaborane. A small amount of pentaborane splashed onto the bare hands of one person who stood close to a damaged drum and breathed in the air close to the drum. Within 15 to 20 minutes, irritation of the respiratory tract, convulsions, muscle weakness and severe metabolic acidosis without respiratory compensation occurred and within 15 to 45 minutes, functional disorders or liver damage developed. The exposed areas of skin, the conjunctivae and the oral mucosa were reddened. Despite rapid emergency medical treatment, the worker died 7 days later of bilateral necrotic pneumonia, fatty degeneration of the liver and widespread degeneration of the brain tissue. In addition, there were no mature sperm in the testes. A second person, who worked in an adjacent building into which pentaborane had drifted, suffered from convulsions, tachycardia and severe metabolic acidosis without respiratory compensation. A biopsy of the liver carried out 15 days later revealed non-specific hepatitis with mild inflammation of the liver lobes. After 6 months, muscle weakness, poor to no coordination and spasms remained, and the CT scan revealed cortical atrophy and ventricular dilation. The person had to remain in hospital. A third exposed person, who worked together with the first person, did not remain in the vicinity of the drum, but left the building. He suffered from muscle twitches, was confused, disoriented and agitated, and had hallucinations. He developed erythema on his face, neck, chest and upper extremities. The neurological examinations did not determine unusual findings, but psychiatric changes were observed. Only mild acidosis occurred. The third patient was discharged after 11 days without sensory-motor deficits (IFA 2002; Yarbrough et al. 1985).

One worker and 13 rescue workers aged 26.3 ± 8.3 years, 7 men and 7 women, were involved in the accident described above. Eleven of them were treated in hospital within 1 hour after exposure. They had conjunctivitis and the exposed areas of skin were reddened. Ten of the exposed persons who were interviewed on the following day reported dizziness, blurred vision and tiredness, 5 of them suffered from myoclonic jerks, 4 had hallucinations and 3 suffered from memory loss. The EEG of 7 of the 10 exposed persons revealed bitemporal theta waves. This was considered a sign of toxic encephalopathy. Neurological and neuropsychological examinations, a dexamethasone suppression test, lumbar puncture, EEG, CT scan of the head with determination of the ventricular brain ratio (VBR), and blood and urine analyses were carried out 1 to 3 months after exposure. At that time, no unusual findings were detected in the EEG of the 11 persons who had been examined immediately after exposure. One patient who underwent the first CT scan 3 months after exposure was found to have grade 1 dysrhythmia in the left temporal lobe. Anomalies were detected in the CT scans of 6 of 12 patients 2 months after exposure. The mean VBR of the exposed persons differed significantly from that of the control persons of a similar age. Reduced performance was determined in 5 of 11 neuropsychological tests, for example in short-term memory tests, sustained attention tests and in the block design test. The changes in the neuropsychological tests were regarded as substance-related because, overall, the CT scans and the Hopkins Symptom Checklist for emotional topics were in the normal range (Hart et al. 1984). The 2 reports cannot be used for a quantitative evaluation because the substances in the air at the accident site were not determined and the levels of exposure were not reported. However, the symptoms are typical of exposure to pentaborane.

Another study described neuropsychological examinations that were carried out in the same cohort 4 to 12 weeks after exposure. Physical and neurological parameters and the routine laboratory analyses were normal. Of the persons in the examined cohort, 64% still suffered from lethargy, 57% had difficulties concentrating, 57% had poor emotional control, 50% had sleep disorders and 43% suffered from nightmares that were related to the accident. The CNS damage was manifest as psychiatric symptoms, neuropsychological deficits, changes in the EEG, elevated neurotransmitter levels in the CNS and findings in the CT scan. Posttraumatic stress was observed in 7 patients and mild brain dysfunction was found in another 7 patients. A statistically significant relationship between neuropsychological test results and psychiatric diagnoses was not established (Silverman et al. 1985).

## 5 Animal Experiments and in vitro Studies

No further data from animal studies have become available since the documentation for pentaborane was published in 1974. Therefore, only the long-term studies relevant for the derivation of a MAK value and the short-term studies that were included in these publications are reviewed again.

### 5.1 Acute toxicity

#### 5.1.1 Inhalation

The LC_50_ values of pentaborane were found to be between 5 ml/m^3^ (4 hours) and 392 ml/m^3^ (0.5 minutes) for mice (no other details), between 10.4 ml/m^3^ (60 minutes) and 66.6 ml/m^3^ (5 minutes) for “white” rats (no other details) and between 34.9 ml/m^3^ (15 minutes) and 278.9 ml/m^3^ (2 minutes) for dogs (no other details); the animals died within 24 hours. Pentaborane did not cause mortality in monkeys (no other details) up to a concentration of 139.8 ml/m^3^ (2 minutes); however, convulsions and tremor occurred. Restlessness, tremor, ataxia, convulsions and running movements of the extremities were observed. Mice reacted with greater sensitivity than monkeys and the latter reacted with greater sensitivity than dogs. The effects were observed mainly in the CNS; no gross-pathological findings were obtained in the other organs (Henschler 1974; Weir et al. 1964).

#### 5.1.2 Oral administration

There are no data available.

#### 5.1.3 Dermal application

No signs of toxicity were observed in dogs (no other details) after a single application to the skin of the whole body with the exception of the head. The shaved skin of the dogs was exposed to concentrations of 580 ml/m^3^ for 2 hours, concentrations of 550 ml/m^3^ for 4 hours or concentrations of 710 ml/m^3^ for 6 hours. The response time in learning tests was prolonged 1 to 2 hours after exposure and the elimination of boric acid with the urine was increased by a factor of 5 (Henschler 1974; Weir et al. 1964).

### 5.2 Subacute, subchronic and chronic toxicity

#### 5.2.1 Inhalation

Beagle dogs were exposed whole-body to pentaborane in various concentrations for 2 to 3 days: 3.7 ml/m^3^ (n = 3) for 60 minutes a day, 10.2 ml/m^3^ (n = 4) for 15 minutes a day or 19.8 ml/m^3^ (n = 4) for 5 minutes a day. The behaviour of the dogs was examined in a shock avoidance test 1 hour, 2 hours and 24 hours after every exposure. On day 1, no unusual findings were noticeable in the animals; changes in behaviour and convulsions were observed from day 2 onwards. The third exposure to 3.7 ml/m^3^ was stopped early because one animal collapsed during exposure and one of the 3 animals died. Other findings were lethargy, small haemorrhages on the iris or scleral injections in the eyes. In behavioural tests, the response times of the animals were delayed and the animals refused to jump. Additional findings were reported, but a no-effect concentration was not determined (Henschler 1974; Weir et al. 1964). This study has not been reviewed in more detail because the low concentration was not the lowest adverse effect concentration.

Groups of 30 rats, 20 hamsters, 12 rabbits, 4 dogs and 2 monkeys were exposed together in an exposure chamber to a pentaborane concentration of 0.2 ml/m^3^ for 6 hours a day on 5 days a week for 6 months (see Table 1). Restlessness, tremor, ataxia, convulsions and muscle twitches were observed in dogs and monkeys. Rabbits, dogs and monkeys consumed less food and some of them less water, leading to reduced body weight gains. Mortality was observed in these species and in rats. Discharge from the nose and eyes was observed in rats and rabbits. This suggests irritation caused by the substance. The results of the bromsulphthalein test showed increased levels in dogs, which is a sign of impaired liver function. Other effects on the kidneys or substance-specific histopathological changes in the examined organs were not reported (Henschler 1974; Levinskas et al. 1958).

**Tab. 1:** Toxicity of pentaborane after repeated inhalation exposure (Levinskas et al. 1958)

**Species,** **strain,** **number per group**	**Exposure^a)^**	**Findings^b)^**
mice, CFW, 11 ♂; rats, CFW, 12 ♂; guinea pigs, albino, 2 ♂; rabbits, New Zealand White, 6 ♂	4 weeks, 1.0 ml/m^3^, 6 hours/day, 5 days/week	cumulative mortality: mice: day 3: 1/11, day 5: 2/11, day 10: 4/11, day 12: 8/11, day 13: 9/11; rabbits: day 9: 1/6, day 10: 3/6, day 12: 6/6; guinea pigs: day 10: 2/2; rats: day 12: 9/12; signs of irritation: guinea pigs: thick yellow nasal discharge; rats: clear to bloody discharge from the nose; body weights reduced in comparison with the initial weights: 5% in rats, 12% in rabbits, 23% in guinea pigs
rats, CFW, 30 ♂, additional animals for examinations every 2 weeks	6 months, 0.2 ml/m^3^, 6 hours/day, 5 days/week	mortality: 6/30, lethargy, reddish discharge from the nose, absence of grooming, haematocrit ↑, urine: no increase in phenolsulfonphthalein retention, liver: organ weights and glycogen levels not affected; **from 3.5 months**: reddish-brown discharge from the eyes and nose, body weight losses, mortality: 6/30 presumably resulting from a bacterial infection caused by new animals in the cage
golden hamsters, 20 ♂	6 months, 0.2 ml/m^3^, 6 hours/day, 5 days/week	mortality: 3/20, intermittent short-term, mild lethargy
rabbits, New Zealand White, 12 ♂	6 months, 0.2 ml/m^3^, 6 hours/day, 5 days/week	mortality: 4/12, discharge from the eyes and nose, feed and water consumption ↓, body weight gains ↓, ruffled fur
dogs, mongrel, 4 ♀	6 months, 0.2 ml/m^3^, 6 hours/day, 5 days/week	mortality: 1/4, feed consumption ↓, vomiting, apathy, hypaesthesia, anaesthesia, mobility of the hind legs ↓, tremor, incoordination, body weight gains ↓, slower excretion of intravenously injected bromsulphthalein with the urine after 20 minutes and 45 minutes (sign of impaired liver function)
monkeys, rhesus, 2 ♂	3 weeks, 0.2 ml/m^3^, 6 hours/day, 5 days/week	mortality: day 4: 1/2, day 15: 1/2 sacrificed in extremis, feed consumption ↓, vomiting, apathy, hypaesthesia, anaesthesia, mobility of the hind legs decreased up to paralysis, tremor, coordinated movements no longer possible, slight improvement of the symptoms at the weekends

^a)^ all animals exposed together in an exposure chamber

^b)^ no data whether there was statistical significance

**Summary**: A no-effect concentration cannot be estimated because even the lowest pentaborane concentration tested of 0.2 ml/m^3^ was lethal for rats, hamsters, rabbits and dogs within the 6-month exposure period and for both monkeys after 3 weeks.

#### 5.2.2 Oral administration

There are no data available.

#### 5.2.3 Dermal application

There are no data available.

### 5.3 Local effects on skin and mucous membranes

There are no studies available that specifically investigated the effects of the substance on skin and mucous membranes.

Nasal discharge was reported in rats and rabbits exposed to a pentaborane concentration of 0.2 ml/m^3^ for 6 months (see Section 5.2.1). This was interpreted as a reaction to irritation. These effects were not observed in monkeys, dogs or hamsters after exposure according to the same protocol (Levinskas et al. 1958).

### 5.4 Allergenic effects

There are no data available.

### 5.5 Reproductive and developmental toxicity

There are no data available.

### 5.6 Genotoxicity

There are no data available.

### 5.7 Carcinogenicity

There are no data available.

## 6 Manifesto (MAK value/classification)

The critical effect of pentaborane is its toxic effects on the CNS. Marked effects were observed at concentrations below 1 ml/m^3^ in humans and at 0.2 ml/m^3^ in various animal species.

**MAK value and peak limitation. **According to earlier reports, pentaborane has toxic effects on the CNS in humans “below 1 ml/m^3^” (Mindrum 1964). Likewise, animal studies carried out in 1958 reported effects on the CNS after the exposure of rats, dogs and monkeys for 6 months to the only concentration tested of 0.2 ml/m^3^; furthermore, mortality occurred in these species and in hamsters and rabbits (Levinskas et al. 1958). As the available studies do not provide evidence of a possible NOAEC (no observed adverse effect concentration), a health-based MAK value cannot be derived for pentaborane according to the criteria currently valid; the earlier MAK value has therefore been withdrawn and the substance is listed in Section II b of the List of MAK and BAT Values. Pentaborane is accordingly not classified in any of the peak limitation categories.

**Prenatal toxicity. **There are no studies available for this end point. As it is not possible to derive a MAK value, pentaborane is not classified in any of the pregnancy risk groups.

**Germ cell mutagenicity and carcinogenicity. **No studies or evidence of germ cell mutagenicity or carcinogenicity are available. Pentaborane is therefore not classified in any of the germ cell mutagen or carcinogen categories.

**Absorption through the skin. **Exposure to liquid pentaborane is unlikely because of its high vapour pressure. Models cannot be used to calculate absorption through the skin because pentaborane decomposes in water. On the basis of a study in dogs, the amount absorbed through the skin from the gaseous phase is estimated to be at the most 1% of the amount absorbed by inhalation. Therefore, pentaborane has not been designated with “H” (for substances which can be absorbed through the skin in toxicologically relevant amounts).

**Sensitization. **There are no findings available for the sensitizing effects of pentaborane in humans or any data from animal studies or new approach methods (NAMs). Pentaborane has therefore not been designated with either “Sh” or “Sa” (for substances which cause sensitization of the skin or airways).
